# Phase 2 study of lenvatinib in patients with advanced hepatocellular carcinoma

**DOI:** 10.1007/s00535-016-1263-4

**Published:** 2016-10-04

**Authors:** Kenji Ikeda, Masatoshi Kudo, Seiji Kawazoe, Yukio Osaki, Masafumi Ikeda, Takuji Okusaka, Toshiyuki Tamai, Takuya Suzuki, Takashi Hisai, Seiichi Hayato, Kiwamu Okita, Hiromitsu Kumada

**Affiliations:** 1 Department of Hepatology, Toranomon Hospital, Toranomon 2-2-2, Minato-ku, Tokyo, 105-8470 Japan; 2 Department of Gastroenterology and Hepatology, Kinki University School of Medicine, Osaka, Japan; 3 Department of Hepatobiliary and Pancreatology, Saga-Ken Medical Centre KOSEIKAN, Saga, Japan; 4 Department of Gastroenterology and Hepatology, Osaka Red Cross Hospital, Osaka, Japan; 5 Department of Hepatobiliary and Pancreatic Oncology, National Cancer Center Hospital East, Kashiwa, Japan; 6 Department of Hepatobiliary and Pancreatic Oncology, National Cancer Center Hospital, Tokyo, Japan; 7 Eisai Co., Ltd.,, Tokyo, Japan; 8 Shunan Memorial Hospital, Yamaguchi, Japan

**Keywords:** Hepatocellular carcinoma, Lenvatinib, Tyrosine kinase inhibitor, E7080, Vascular endothelial growth factor inhibitor

## Abstract

**Background:**

Lenvatinib is an oral inhibitor of vascular endothelial growth factor receptor 1–3, fibroblast growth factor receptor 1–4, platelet-derived growth factor receptor alpha, RET, and KIT. This phase 2, single-arm, open-label multicenter study evaluated lenvatinib in advanced hepatocellular carcinoma (HCC).

**Methods:**

Patients with histologically/clinically confirmed advanced HCC who did not qualify for surgical resection or local therapies received lenvatinib at a dosage of 12 mg once daily (QD) in 28-day cycles. The primary efficacy endpoint was time to progression (TTP) per modified Response Evaluation Criteria in Solid Tumors v1.1; secondary efficacy endpoints included objective response rate (ORR), disease control rate (DCR), and overall survival (OS).

**Results:**

Between July 2010 and June 2011, 46 patients received lenvatinib at sites across Japan and Korea. The median TTP, as determined by independent radiological review, was 7.4 months [95 % confidence interval (CI): 5.5–9.4]. Seventeen patients (37 %) had partial response and 19 patients (41 %) had stable disease (ORR: 37 %; DCR: 78 %). Median OS was 18.7 months (95 % CI: 12.7–25.1). The most common any-grade adverse events (AEs) were hypertension (76 %), palmar-plantar erythrodysesthesia syndrome (65 %), decreased appetite (61 %), and proteinuria (61 %). Dose reductions and discontinuations due to AEs occurred in 34 (74 %) and 10 patients (22 %), respectively. Median body weight was lower in patients with an early (<30 days) dose withdrawal or reduction than in those without.

**Conclusions:**

Lenvatinib 12-mg QD showed clinical activity and acceptable toxicity profiles in patients with advanced HCC, but early dose modification was necessary in patients with lower body weight. Further development of lenvatinib in HCC should consider dose modification by body weight.

**Trial registration ID:**

www.ClinicalTrials.gov NCT00946153.

**Electronic supplementary material:**

The online version of this article (doi:10.1007/s00535-016-1263-4) contains supplementary material, which is available to authorized users.

## Introduction

In hepatocellular carcinoma (HCC), which accounts for 85–90 % of primary liver cancers [1], increased expression of vascular endothelial growth factor (VEGF) levels has been correlated with angiogenic activity, tumor progression, and poor prognosis [2, 3].

Sorafenib is currently the only systemic VEGF-targeted therapy to have demonstrated a survival benefit in patients with advanced HCC [4, 5]. However, the median overall survival (OS) and time to progression (TTP) with sorafenib are only ~1 year and ~4 months, respectively, with frequent dose reductions or discontinuations due to adverse events, including severe skin toxicity [6–8]. Therefore, there is still an unmet need for better therapeutic options for patients with advanced HCC. To date, phase 3 trials of several agents, including sunitinib, brivanib, and linifanib, have failed to demonstrate benefit in advanced HCC [6].

Lenvatinib—an oral, multi-tyrosine kinase receptor inhibitor of VEGF receptors 1–3, fibroblast growth factor (FGF) receptor 1–4, platelet-derived growth factor (PDGF) receptor alpha, and KIT and RET proto-oncogenes [9–11]—was approved for radioiodine-refractory differentiated thyroid cancer at a dose of 24 mg once daily (QD) in 28-day cycles [12]. However, for the clinical development of therapeutics in HCC, re-evaluation of the starting dose of any investigational agent is recommended [13]. In a phase 1 study of lenvatinib in HCC, the maximum tolerable dose in patients with HCC and Child Pugh (CP) class A liver function was 12 mg QD [14]. This dose also showed blood trough concentrations comparable to those with the 25-mg QD dose determined to be the maximum tolerated dose in solid tumors [15] with preliminary evidence of tumor shrinkage. Here we assessed the antitumor activity and safety of lenvatinib in this phase 2 study in patients with advanced HCC.

## Methods

### Patients

Patients ages 20–80 years had clinically confirmed advanced HCC with residual disease not qualifying for surgical resection or local therapies, including transarterial chemoembolization; ≥1 measurable target lesion by Response Evaluation Criteria in Solid Tumors version 1.1 (RECIST v1.1); [16] 1–3 tumor lesions >3 cm in diameter (>5 cm diameter if only one lesion) or four or more lesions or portal vein invasion, extrahepatic invasion; Eastern Cooperative Oncology Group performance status of 0 or 1; CP score of 5 or 6 (CP class A); platelet count ≥50 × 10^9^/L, absolute neutrophil count ≥1.5 × 10^3^/μL; aspartate transaminase and alanine transaminase ≤5.0 times the upper limit of normal; and serum creatinine ≤2.0 mg/dL or calculated creatinine clearance ≥40 mL/min. Patients had to have had a hepatectomy and local therapy for HCC at least 6 and 4 weeks, respectively, prior to study enrollment. Exclusion criteria included clinically symptomatic brain metastasis or meningeal carcinomatosis; receipt of ≥1 systemic chemotherapy, including targeted therapy or transarterial infusion chemotherapy; QT-corrected interval by the Fredericia method >500 ms at screening; mean blood pressure ≥150/90 mmHg; presence of a progressive central nervous system disease; or a clinically significant hemorrhagic or thrombotic event within 4 weeks prior to study enrollment.

### Study design and treatment

Patients in this single-arm, open-label multicenter study of lenvatinib monotherapy for advanced HCC (NCT00946153) received daily oral administration of 12 mg lenvatinib (12-mg QD) in 28-day cycles until disease progression, unacceptable toxicity, or withdrawal of consent. Dose interruption and sequential reduction of lenvatinib (to 8- and 4-mg QD) were permitted for drug-related adverse events (AEs; see Online Resource Table S1). Once reduced, the dose could not be re-escalated. The study drug was discontinued if patient recovery time was >2 weeks.

This study was conducted in accordance with local laws, the Declaration of Helsinki, and International Conference on Harmonization Good Clinical Practice guidelines, and with the approval of each institutional review board. All patients provided written informed consent.

### Study assessments

The primary efficacy endpoint was TTP per modified RECIST (mRECIST; modified to evaluate viable lesions) [17] by an independent radiologic review committee (IRRC). mRECIST uses viable target lesions in dynamic computed tomography and is suitable for the assessment of tyrosine kinase inhibitors in HCC [17, 18]. Secondary efficacy endpoints included objective response rate (ORR), disease control rate (DCR), and OS. Tumor response was evaluated every 8 weeks using mRECIST v1.1 by both the IRRC and study investigators. An exploratory analysis to reassess tumors using RECIST v1.1 was also performed by the IRRC. Safety was assessed by physical examinations, clinical and laboratory evaluations, vital signs, and electrocardiograms. AEs were graded according to the National Cancer Institute Common Terminology Criteria for AEs, version 3.0. A pre-dose blood sample was obtained for pharmacokinetic (PK) assessment on days 1, 8, 15, and 22 of cycle 1, and day 1 of cycles 2 and 3, using a validated liquid chromatography–tandem mass spectrometry method [19].

### Statistical considerations

Time-to-event endpoints, including TTP and OS, were summarized using Kaplan–Meier estimates. The sample size of 46 patients was based on an 80 % probability that the lower limit of the two-sided 90 % confidence interval (CI) of median TTP would exceed the threshold of 2.7 months (based on the Sorafenib HCC Assessment Randomized Protocol trial [20]), with an expected median TTP of 4.1 months for lenvatinib. Sample size determination also assumed exponential distribution for TTP, 12 months of enrollment, 6 months of follow-up, and a 10 % patient exclusion/dropout rate. An interim evaluation was conducted when 21–23 patients became evaluable for the 2-month tumor assessment. If the number of patients with progressive disease within 2 months was ≥10 (≥80 % Bayesian posterior probability for proportion of patients with progressive disease at 2 months ≥35 %), then study discontinuation would be considered due to futility. Follow-up was continued until final analysis was performed when 67 % of patients had died.

## Results

### Patient characteristics

Overall, 46 patients were enrolled and received lenvatinib at 14 sites across Japan and Korea between July 2010 and June 2011. All patients were included in the safety and efficacy analyses. Patient demographics and baseline characteristics are listed in Table 1.

**Table 1:** Patient demographics and baseline characteristics

Characteristic	Patients (*N* = 46)
Median age, years (range)	66.5 (37–80)
Sex, *n* (%)	
Female	13 (28.3)
Male	33 (71.7)
Region, *n* (%)	
Japan	43 (93.5)
South Korea	3 (6.5)
Median weight, kg (range)	56.7 (42.8–85.5)
ECOG PS, *n* (%)	
0	38 (82.6)
1	8 (17.4)
Child Pugh Class, *n* (%)	
A	45 (97.8)
B	1 (2.2)
BCLC staging*, n* (%)	
B	19 (41.3)
C	27 (58.7)
Portal vein invasion, *n* (%)	
Yes	5 (10.8)
No	41 (89.1)
Extrahepatic metastasis, *n* (%)	
Yes	21 (45.7)
No	25 (54.3)
Cause of HCC, *n* (%)	
Hepatitis B	15 (32.6)
Hepatitis C	27 (58.7)
Alcohol	2 (4.3)
Non-alcohol-related fatty liver disease	1 (2.2)
Unknown	2 (4.3)
AFP value at baseline^a^	
<200 ng/mL	27 (57.7)
≥200 ng/mL	18 (39.1)
Prior surgery for HCC, *n* (%)	
No	27 (58.7)
Yes	19 (41.3)
Prior local therapy, *n* (%)	
No	4 (8.7)
Yes	42 (91.3)
RFA	32 (69.6)
PEI	12 (26.1)
TACE	39 (84.8)
TAE	3 (6.5)
Prior chemotherapy, *n* (%)	
Sorafenib	6 (13.0)
Other systemic chemotherapy	5 (10.9)
Hepatic intra-arterial chemotherapy	5 (10.9)

*BCLC* Barcelona Clinic Liver Cancer, *ECOG-PS* Eastern Cooperative Oncology Group Performance Status, *HCC* hepatocellular carcinoma, *AFP* alpha-fetoprotein, *PEI* percutaneous ethanol injection, *RFA* radiofrequency ablation, *TACE* transcatheter arterial chemoembolization, *TAE* transarterial embolization

^a^AFP data were unavailable for one patient

### Efficacy

**Fig. 1:**
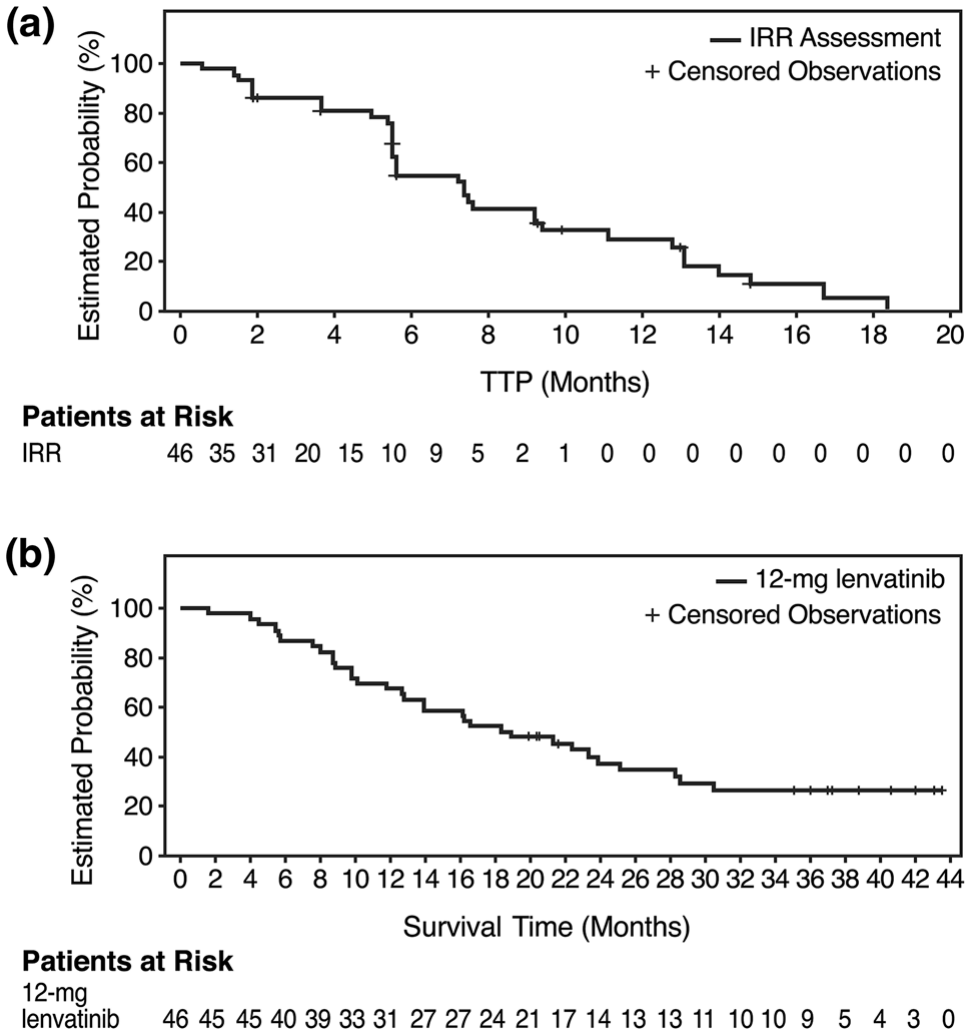
Kaplan–Meier estimates of **a** TTP and **b** OS. Median TTP was 7.4 months as assessed by an IRRC comprising four independent radiologists. Median OS was 18.7 months. *IRRC* independent radiologic review committee, *TTP* time to progression

Median TTP was 7.4 months (95 % CI: 5.5–9.4) as assessed by IRRC per mRECIST (Fig. 1a). Median TTP was 12.8 months (95 % CI: 7.2–14.7) by investigator assessment. Seventeen patients (37 %) achieved a partial response and 19 patients (41 %) had stable disease ≥8 weeks, with a DCR of 78 % by IRRC (Table 2). Outcomes using RECIST v1.1 criteria are also provided in Table 2. Median OS was 18.7 months (95 % CI: 12.7–25.1; Fig. 1b).

**Table 2:** Tumor responses

Response category	Investigator assessment (mRECIST), *n* = 46	IRRC assessment (mRECIST), *n* = 46	IRRC assessment (RECIST 1.1), *n* = 46
Best response, *n* (%)			
Complete response	0 (0)	0 (0)	0 (0)
Partial response	17 (37)	17 (37)	11 (24)
Stable disease	21 (46)	19 (41)	25 (54)
Progressive disease	5 (11)	6 (13)	6 (13)
Not evaluable	3 (7)	4 (9)	4 (9)
Objective response rate, *n* (%)	17 (37)	17 (37)	11 (24)
Disease control rate, *n* (%)	38 (83)	36 (78)	36 (78)

*IRRC* independent radiologic review committee, *mRECIST* modified response evaluation criteria in solid tumors

**Fig. 2:**
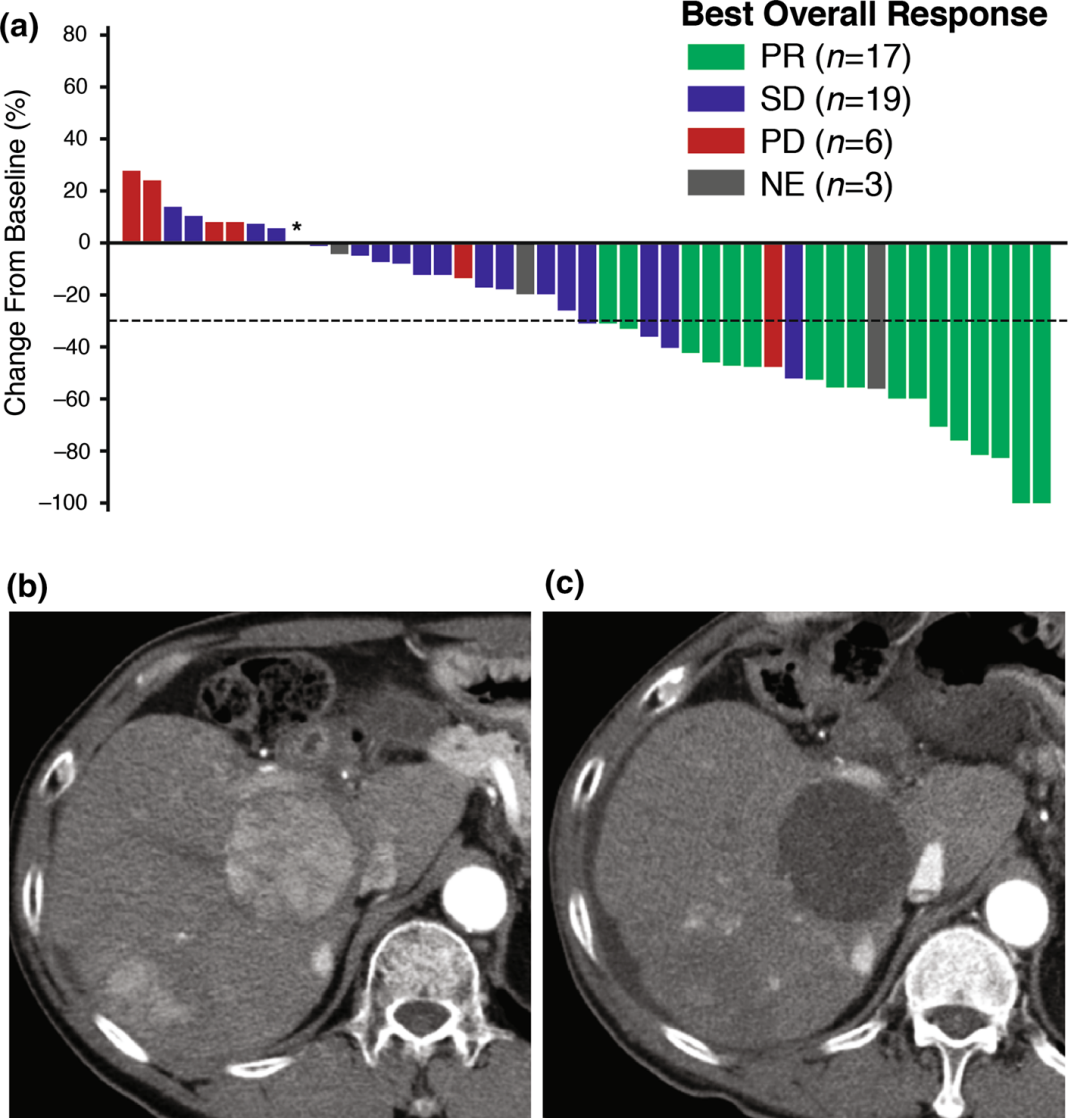
**a** Waterfall plot of changes in tumor size by IRRC assessment. One patient was excluded from the plot due to lack of IRRC assessment of target legion at baseline. The patient marked with an asterisk showed a best overall response of SD. **b** Representative liver lesion of HCC at baseline on arterial phase CT. **c** Representative liver lesion on arterial phase CT after 1 year of lenvatinib treatment. *CT* computed tomography, *HCC* hepatocellular carcinoma, *IRRC* independent radiologic review committee, *PD* progressive disease, *PR* partial response, *SD* stable disease, *NE* not evaluable

Tumor reduction of target lesions, assessed by IRRC, occurred in 80 % of patients (Fig. 2a–c). Subgroup analyses indicated that lenvatinib clinical activity was maintained regardless of tumor status (with or without extrahepatic spread or portal vein invasion), type of underlying hepatitis [hepatitis B virus (HBV) or hepatitis C virus], receipt of previous chemotherapy, or alpha-fetoprotein levels (AFP; <200 ng/mL or ≥200 ng/mL; see Online Resource Table S2). On the other hand, AFP levels may affect the prognosis for patients with HCC (Online Resource Figure S1).

### Safety

Median and mean durations of lenvatinib treatment were 7.3 and 9.0 months, respectively. All 46 patients experienced at least one AE. The most common any-grade AEs (Table 3) were hypertension (76 %), palmar-plantar erythrodysesthesia syndrome (PPES; 65 %), decreased appetite (61 %), and proteinuria (61 %). The incidence of serious AEs (SAEs) was 48 %, and the most frequently reported SAE was hepatic encephalopathy (11 %). No treatment-related deaths were reported. Two patients died within 30 days of receiving their last dose of lenvatinib—one from pneumonia and one from liver tumor rupture.

**Table 3:** Common adverse events occurring in ≥20 % of patients

Adverse event	Any grade, *n* = 46	Grade 3, *n* = 46	Grade 4, *n* = 46
Hypertension	35 (76.1)	25 (54.3)	0
Palmar-plantar erythrodysesthesia syndrome	30 (65.2)	4 (8.7)	0
Decreased appetite	28 (60.9)	1 (2.2)	0
Proteinuria	28 (60.9)	9 (19.6)	0
Fatigue	25 (54.3)	0	0
Diarrhea	20 (43.5)	6 (13.0)	0
Constipation	19 (41.3)	0	0
Nausea	17 (37.0)	1 (2.2)	0
Dysphonia	17 (37.0)	0	0
Thrombocytopenia	16 (34.8)	9 (19.6)	1 (2.2)
Peripheral edema	16 (34.8)	0	0
Decreased weight	14 (30.4)	2 (4.3)	0
Neutropenia	13 (28.3)	2 (4.3)	0
Nasopharyngitis	13 (28.3)	0	0
Rash	13 (28.3)	0	0
Increased blood thyroid-stimulating hormone level	12 (26.1)	0	0
Back pain	11 (23.9)	0	0
Stomatitis	11 (23.9)	0	0
Vomiting	11 (23.9)	1 (2.2)	0
Pyrexia	10 (21.7)	0	0
Hypothyroidism	10 (21.7)	0	0
Insomnia	10 (21.7)	0	0

**Fig. 3:**
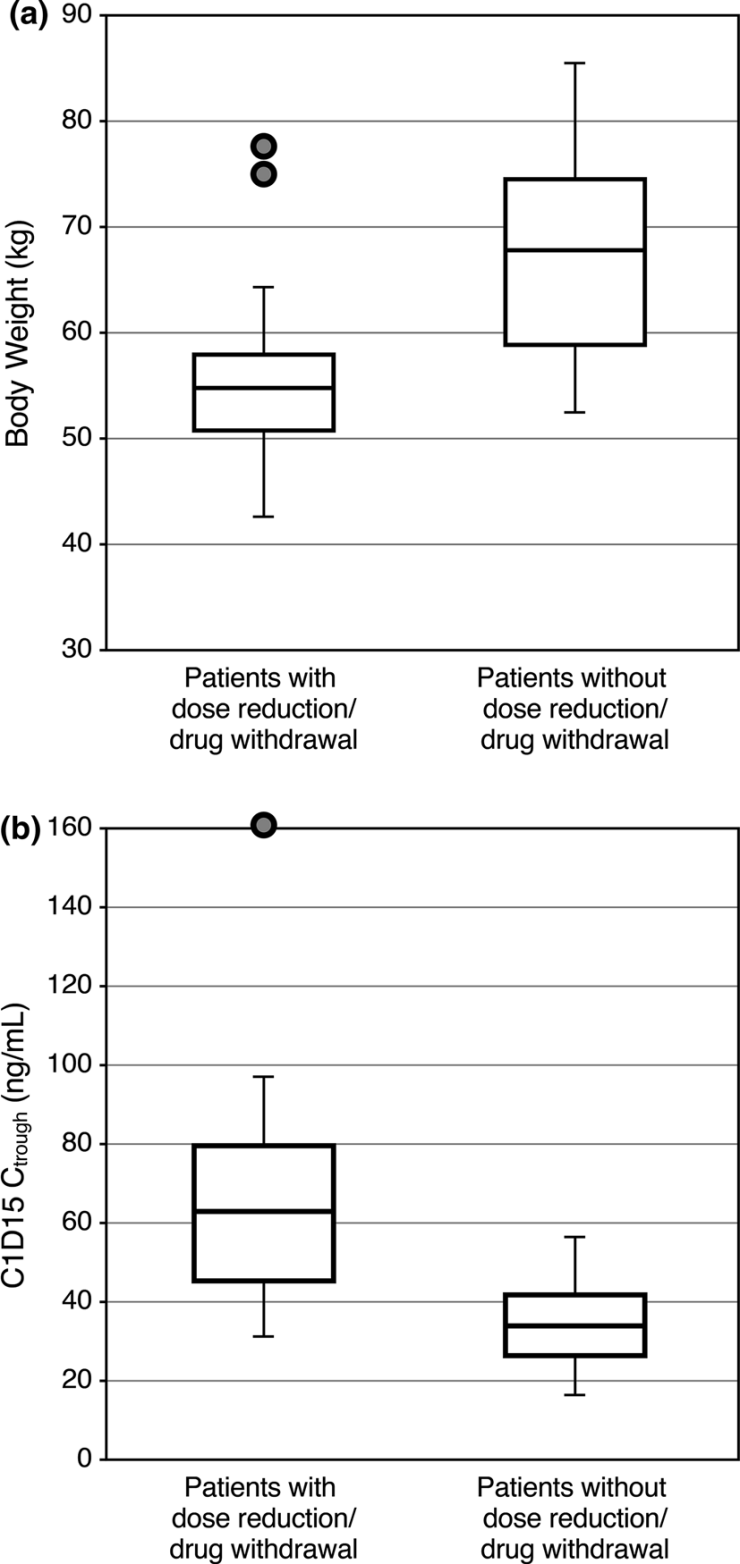
**a** *Boxplot* of body weight for patients with vs. without adverse events that led to dose reduction or dose withdrawal within 30 days of lenvatinib treatment initiation. **b** *Boxplot* of lenvatinib C_trough_ level 15 days after lenvatinib treatment initiation for patients with vs. without adverse events that led to dose reduction or withdrawal within 30 days. *C1D15* cycle 1 day 15, *C*_trough_ minimum concentration of lenvatinib

AEs were generally manageable with dose modifications. Lenvatinib dose reductions due to AEs occurred in 34 patients (74 %). Ten patients (22 %) discontinued study treatment due to toxicity. The most frequently reported AE leading to study drug withdrawal was proteinuria (11 %). Twenty-two patients (48 %) experienced AEs leading to dose withdrawal or dose reduction <30 days after starting lenvatinib. In an exploratory analysis of differences in baseline characteristics between patients who did and did not require an early dose withdrawal or reduction, body weight and minimum concentration of lenvatinib (C_trough_) were identified as potential differentiators (Fig. 3). Median body weight was lower for patients who experienced an early dose withdrawal or reduction (54.1 kg) than for those who did not (67.6 kg) (Fig. 3a). The median C_trough_ values on cycle 1 day 15 (C1D15) in patients with and without dose modifications were 62.4 and 33.9 ng/mL, respectively (Fig. 3b). The Spearman correlation coefficient between body weight and lenvatinib C_trough_ at C1D15 was −0.64.

## Discussion

Multi-tyrosine kinase inhibitors have shown limited success in advanced HCC, with reported TTP ranging from 2.8 to 5.4 months and ORR from 6.9 to 9 % [4, 5, 7, 21, 22]. In this study, lenvatinib 12-mg QD showed promising clinical activity in patients with advanced HCC, with a median TTP of 7.4 months as assessed by IRRC (12.8 months by investigator). Lenvatinib also demonstrated tumor shrinkage in 80 % of patients, with a response rate of 37 % per mRECIST and 24 % per conventional RECIST.

Although progression-free survival is the preferred OS surrogate endpoint in most solid tumor trials, it is a particularly unreliable endpoint in HCC studies, because death from the natural history of cirrhosis may confound results; TTP is therefore the recommended endpoint for early-stage trials of HCC [13]. In HCC, response rates derived from mRECIST have been shown to better correlate with OS than those from conventional RECIST [18]. Notably, despite a similarity in best overall responses between investigator and independent assessments, the median TTP was substantially different, suggesting a bias by the investigators in determining the timing of disease progression, ostensibly so that their patients could continue therapy.

The median OS in this study was 18.7 months. Although subgroup analyses indicated that median TTP was comparable regardless of baseline AFP levels, OS was longer in the 61 % of patients with lower vs. higher AFP levels (23.5 vs. 13.3 months, respectively). Therefore, it is possible that the long median OS observed in this study may have been driven by those patients with lower baseline AFP levels, because elevated AFP levels are associated with an increased mortality rate in HCC [23]. Other risk factors examined included extrahepatic spread and HBV. In this study, lenvatinib activity was observed even in these patients with poor prognoses; however, this conclusion is limited by the small numbers of patients in each subgroup. Another limitation is the single-arm design of the study, and the possibility that results may be influenced by patient selection. However, we note that patient characteristics in this study were typical of the population of patients with HCC who required sorafenib therapy in Japan [8, 24].

The most common AEs in this study included hypertension, PPES, decreased appetite, proteinuria, and fatigue, which are well-known class effects of VEGF-targeted therapies and are consistent with the known safety profile of lenvatinib. Although the incidence of grade 3 hypertension was high (54 %), this included patients whose blood pressure was controlled by two or more antihypertensive drugs. No patient required a dose modification or discontinuation due to hypertension; therefore, it was considered to be manageable. Although 65 % of patients experienced PPES, the incidence of grade 3 PPES was only 9 %. The incidence of grade 3 or 4 thrombocytopenia was also high (22 %); however, in all but one patient who discontinued lenvatinib, thrombocytopenia was controllable with dose modifications. There was no report of grade 3 or higher bleeding related to the study drug. Although hepatic encephalopathy was the most common SAE (five patients) in this study, all five patients also had constipation, and three had dehydration—known risk factors for hepatic encephalopathy [25]. These events were managed with dose modifications as well as treatment of constipation and dehydration.

Dose reduction occurred frequently and early in the course of treatment. Because 74 % of patients required a dose reduction due to AEs, we examined possible risk factors for the development of intolerable toxicities. The influence of body weight on the pharmacodynamics of antiangiogenic agents and resultant toxicity patterns is still uncertain [26]. Additionally, increased lenvatinib exposure was found in patients with severe hepatic impairment [27]. It is possible that in patients with HCC, who typically have impaired hepatic function, lenvatinib PK is more affected by body weight than in healthy individuals or patients with other cancers. Furthermore, careful evaluation of the balance between efficacy and toxicity is especially important in studies of patients with HCC, because high toxicity is a common reason for failure of phase 3 clinical trials in this therapeutic area [6]. Therefore, although drug-related AEs were manageable with dose modifications, and there were no drug-related deaths in this study, lenvatinib exposure was observed to be influenced by body weight in patients with HCC, and adjustment of the starting dose of lenvatinib by body weight in further clinical development of lenvatinib in HCC is recommended.

In conclusion, the administration of lenvatinib 12-mg QD showed clinical activity and acceptable toxicity profiles in patients with advanced HCC, although early dose modification was necessary for the management of toxicities in patients with lower body weight. Based on these findings, a phase 3 study of lenvatinib in HCC is underway, with planned doses of 8 mg in patients with lower body weight (<60 kg) and 12 mg in those with higher body weight (≥60 kg; NCT01761266) [28].

## Electronic supplementary material

Below is the link to the electronic supplementary material.

Supplementary material 1 (PDF 219 kb)

Supplementary material 2 (PDF 242 kb)

Supplementary material 3 (TIFF 1217 kb)
