# Supplementary material for: Phase 2 study of lenvatinib in patients with advanced hepatocellular carcinoma
Source: J Gastroenterol. 2016 Oct 4;52(4):512–9. doi: 10.1007/s00535-016-1263-4 (PMC5357473; doi:10.1007/s00535-016-1263-4)
Supplement: Supplementary file 1 — Supplementary material 1 (PDF 219 kb) [file 535_2016_1263_MOESM1_ESM.pdf]

The Journal of Gastroenterology

Phase 2 Study of Lenvatinib in Patients With Advanced Hepatocellular Carcinoma

Kenji Ikeda, MD,[1] Masatoshi Kudo, MD,[2] Seiji Kawazoe, PhD,[3] Yukio Osaki, MD,[4] Masafumi Ikeda, MD,[5] Takuji Okusaka, MD,[6] Toshiyuki Tamai, MSc,[7] Takuya Suzuki, MSc,[7] Takashi Hisai, MSc,[7] Seiichi Hayato, MSc,[7] Kiwamu Okita, MD,[8] Hiromitsu Kumada, MD[1]

[1]Department of Hepatology, Toranomon Hospital, Tokyo, Japan; [2]Department of Gastroenterology and Hepatology, Kinki University School of Medicine, Osaka, Japan; [3]Department of Hepatobiliary and Pancreatology, Saga-Ken Medical Centre KOSEIKAN, Saga, Japan; [4]Department of Gastroenterology and Hepatology, Osaka Red Cross Hospital, Osaka, Japan; [5]Department of Hepatobiliary and Pancreatic Oncology, National Cancer Center Hospital East, Kashiwa, Japan; [6]Department of Hepatobiliary and Pancreatic Oncology, National Cancer Center Hospital, Tokyo, Japan; [7]Eisai Co., Ltd., Tokyo, Japan; [8]Shunan Memorial Hospital, Yamaguchi, Japan.

Corresponding author:

Kenji Ikeda, MD

Department of Hepatology, Toranomon Hospital

Toranomon 2-2-2, Minato-ku, Tokyo 105-8470, Japan

Email: ikedakenji@tora.email.ne.jp

Telephone: +81-3-3588-1111

Fax: +81-3-3582-7068

Online Resource Table S1: Dose Reduction Guidelines

<table>
<tr><th colspan="3">Drug-related AE Grades</th><th>Dose Delay</th><th>Dose Reduction to Restart</th></tr>
<tr><td colspan="3">HEMATOLOGIC TOXICITIES</td><td></td><td></td></tr>
<tr><td colspan="3">Grade 4</td><td rowspan="2">Delay until ≤ Grade 2</td><td rowspan="2">Decrease 1 dose level[a]</td></tr>
<tr><td colspan="3">Grade 3 thrombocytopenia that requires blood transfusion</td></tr>
<tr><td colspan="3">NONHEMATOLOGIC TOXICITIES</td><td></td><td></td></tr>
<tr><td colspan="3">AST/ALT > 10.0 x ULN</td><td rowspan="7">Delay until ≤ Grade 1 or baseline Grade[b]</td><td rowspan="7">Decrease 1 dose level[a]</td></tr>
<tr><td rowspan="3">Proteinuria</td><td colspan="2">4+ in dipstick</td></tr>
<tr><td rowspan="2">If monitored 3+ proteinuria but not judged grade 3 in dipstick</td><td>Proteinuria >3.5 g for 24 hour urine collection</td></tr>
<tr><td>(Only if urine collection cannot be monitored) with grade ≥ 2 of creatinine</td></tr>
<tr><td colspan="3">Hypertension:
Grade 4 that is uncontrollable despite more intensive therapy than previously</td></tr>
<tr><td colspan="3">Diarrhea/vomiting/nausea:
Grade 3 or higher that is uncontrollable despite maximal supportive therapies</td></tr>
<tr><td colspan="3">Any grade 3–4 nonhematologic toxicity other than above except for abnormal clinical laboratory values that require</td></tr>
</table>

no treatment		

[a]Reduction of lenvatinib dose: 12 mg → 8 mg → 4 mg → discontinue treatment.

[b]Baseline grade is defined as the data from the baseline period.

ALT, alanine aminotransferase; AST, aspartate aminotransferase; ULN, upper limit of normal.
