# Supplementary material for: Phase 2 study of lenvatinib in patients with advanced hepatocellular carcinoma
Source: J Gastroenterol. 2016 Oct 4;52(4):512–9. doi: 10.1007/s00535-016-1263-4 (PMC5357473; doi:10.1007/s00535-016-1263-4)
Supplement: Supplementary file 2 — Supplementary material 2 (PDF 242 kb) [file 535_2016_1263_MOESM2_ESM.pdf]

The Journal of Gastroenterology

Phase 2 Study of Lenvatinib in Patients With Advanced Hepatocellular Carcinoma

Kenji Ikeda, MD,[1] Masatoshi Kudo, MD,[2] Seiji Kawazoe, PhD,[3] Yukio Osaki, MD,[4] Masafumi Ikeda, MD,[5] Takuji Okusaka, MD,[6] Toshiyuki Tamai, MSc,[7] Takuya Suzuki, MSc,[7] Takashi Hisai, MSc,[7] Seiichi Hayato, MSc,[7] Kiwamu Okita, MD,[8] Hiromitsu Kumada, MD[1]

[1]*Department of Hepatology, Toranomon Hospital, Tokyo, Japan;* [2]*Department of Gastroenterology and Hepatology, Kinki University School of Medicine, Osaka, Japan;* [3]*Department of Hepatobiliary and Pancreatology, Saga-Ken Medical Centre KOSEIKAN, Saga, Japan;* [4]*Department of Gastroenterology and Hepatology, Osaka Red Cross Hospital, Osaka, Japan;* [5]*Department of Hepatobiliary and Pancreatic Oncology, National Cancer Center Hospital East, Kashiwa, Japan;* [6]*Department of Hepatobiliary and Pancreatic Oncology, National Cancer Center Hospital, Tokyo, Japan;* [7]*Eisai Co., Ltd., Tokyo, Japan;* [8]*Shunan Memorial Hospital, Yamaguchi, Japan.*

Corresponding author:

Kenji Ikeda, MD

Department of Hepatology, Toranomon Hospital

Toranomon 2-2-2, Minato-ku, Tokyo 105-8470, Japan

Email: ikedakenji@tora.email.ne.jp

Telephone: +81-3-3588-1111

Fax: +81-3-3582-7068

Online Resource Table S2: Efficacy by Subgroups of Interest

**Subgroup**		***n***	**Median, months (95% CI)**	
			**TTP**[a]	**OS**
EHS/portal vein invasion	No	22	7.4 (5.5, 12.8)	17.5 (8.8, 23.4)
	Yes	24	7.4 (3.7, 9.4)	21.5 (9.8, NE)
Hepatitis virus type[b]	HBV	15	9.4 (7.2, 14.8)	22.4 (13.9, 30.5)
	HCV	27	5.6 (5.5, 9.2)	16.2 (9.8, 28.6)
Previous chemotherapy	No	30	5.5 (5.4, 13.1)	20.4 (9.8, 30.5)
	Yes	16	7.6 (5.6, 11.1)	17.8 (11.8, 28.6)
Alpha-fetoprotein (ng/mL)[c]	< 200	27	7.4 (5.5, 11.1)	23.4 (13.9, NA)
	≥ 200	18	7.4 (3.7, 14.0)	13.3 (5.7, 25.1)

[a]Per independent reviewer assessment according to mRECIST

[b]Four patients did not have HBV or HCV

[c]One patient missed AFP sampling at baseline.

CI, confidence interval; EHS, extrahepatic spread; HBV, hepatitis B virus; HCV, hepatitis C virus;

OS, overall survival; TTP, time to progression.
