# Supplementary figures and images for: Phase 2 study of lenvatinib in patients with advanced hepatocellular carcinoma
Source: J Gastroenterol. 2016 Oct 4;52(4):512–9. doi: 10.1007/s00535-016-1263-4 (PMC5357473; doi:10.1007/s00535-016-1263-4)

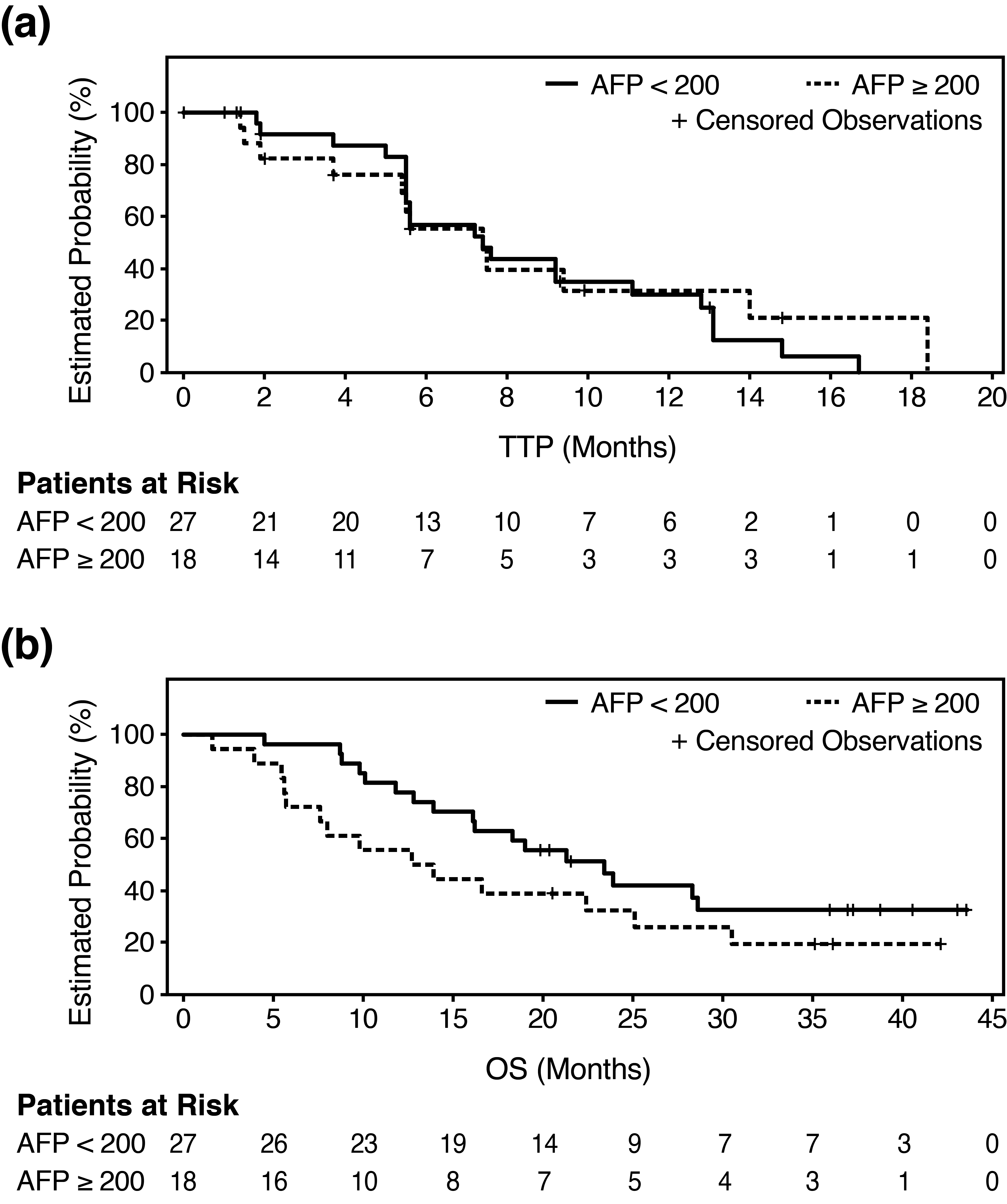

Supplement: Supplementary file 3 — Supplementary material 3 (TIFF 1217 kb) [file 535_2016_1263_MOESM3_ESM.tif]
